# Exploration of Potential Antidepressant Active Ingredients of *Albiziae Flos* via HS–GC–MS, Chemometrics, and Network Pharmacology

**DOI:** 10.1155/jamc/2885034

**Published:** 2026-03-01

**Authors:** Dan Yang, Dan He, Yiwu Wang, Yuan Shen, Jialing Yu, Ruijia Yang, Lin Yang

**Affiliations:** ^1^ College of Pharmacy, Chongqing Medical University, Chongqing, China, cqmu.edu.cn; ^2^ College of Chinese Materia Medica, Chongqing University of Chinese Medicine, Chongqing, 402760, China; ^3^ West China School of Medicine, Sichuan University, Chengdu, China, scu.edu.cn; ^4^ Chongqing Key Laboratory of High Active Traditional Chinese Drug Delivery System, Chongqing Medical and Pharmaceutical College, Chongqing, China, cqmu.edu.cn

## Abstract

*Albiziae Flos* (AF) is a traditional Chinese medicine with an extensive historical background. This study presented an integrated approach combining headspace–gas chromatography–mass spectrometry (HS–GC–MS), chemometrics, and network pharmacology to comprehensively evaluate the volatile components of AF and explore their potential antidepressant mechanisms. A total of 34 volatile compounds were identified through HS–GC–MS analysis. Fingerprint assessment revealed high consistency among 16 batches (similarity: 0.790–0.998), while chemometric analysis successfully discriminated samples from different geographical origins. Network pharmacology screening identified 15 active components and 131 potential targets, revealing multicomponent, multitarget characteristics of AF’s antidepressant effects. Molecular docking simulations demonstrated strong binding affinity between linalool oxide configurations and 12 core targets, primarily through hydrogen bonding and hydrophobic interactions. This work lies in its comprehensive investigation of AF’s volatile components using an integrated analytical–pharmacological approach, providing both a methodological framework for quality assessment and mechanistic insights for antidepressant drug development. Our findings established scientific foundations for quality control of AF and revealed its potential antidepressant mechanisms through multiple pathways.

## 1. Introduction

*Albiziae Flos*​ (AF) is the dehydrated inflorescence or bud of the leguminous plant *Albizia julibrissin Durazz* (AJD), collected on a sunny day or during the summer bud formation and dried promptly. The former is known as “acacia flower,” whereas the latter is termed “acacia rice.” The two differ solely in the selection period, but the effects are similar. AF is a capitulum that has condensed into clusters. The whole pedicel measures 3‐4 cm in length, occasionally separated from the inflorescence, exhibiting a yellow‐green hue, longitudinal orientation, and scanty fur texture. The flowers are densely hairy, slender, and curved, 0.7 ∼ 1 cm long, light yellow or yellowish brown, without pedicels or with few pedicels. It possesses a mild fragrance, is light in texture, exhibits modest astringency, induces a tingling sensation on the tongue, and causes discomfort in the throat [1, 2]. At present, AF is mainly distributed in Jiangxi, Shandong, Guizhou, Zhejiang, and Jiangsu in China [3]. Abroad, AF was mainly produced in warm, arid, and subarid regions such as Brazil and other places [4]. AF has numerous pharmacological properties, and it was first recorded in “Shennong’s Herbal Classic.” Modern pharmacological studies have shown that AF has antianxiety, sedative–hypnotic, antidepressant, hepatoprotective, antibacterial, antioxidant, antiobesity, lowering blood glucose and blood lipids, protecting cardiovascular and gastrointestinal tract, and possessing great potential medicinal value [5]. It was frequently utilized in clinical treatment to alleviate symptoms like restlessness, depression, and insomnia.

Depression and insomnia were a kind of chronic and recurrent mental pathological symptoms. Long‐term insomnia can also accelerate the course of depression. Such diseases often have adverse effects on patients′ physical and mental health and interpersonal communication [6]. The World Health Organization forecasts that depression will emerge as the second most prevalent chronic disease following ischemic heart disease, with the financial burden associated with depression in Western nations anticipated to rank highest by 2030 [7]. The current treatment of depression primarily relies on chemical medicine, such as tricyclic antidepressants, monoamine oxidase inhibitors, tetracyclic antidepressants, selective serotonin reuptake inhibitors (SSRIs), and other drugs [8]. Traditional Chinese medicine (TCM) has multicomponents, multitargets, and multipharmacological synergistic effects [9]. The theories of TCM hold that AF can effectively alleviate depression. In the study of modern clinical efficacy, Jieyu Hehuan Decoction [10], Hehuanle Granules [11], Hehuan Particles [12], and other TCM preparations use AF as the monarch drug. After clinical experiments, the mental state of patients with depression was improved and the recurrence rate was decreased after the treatment of depression with AF.

AF contains a variety of chemical components. It was reported that volatile oil components, flavonoids, and other compounds were detected in Jiangsu and Guangdong [13]. Some scholars used high‐performance liquid chromatography (HPLC), ultra‐high‐performance liquid chromatography–tandem mass spectrometry (UPLC–MS/MS), and other methods to analyze the nonvolatile flavonoids such as quercitrin, isoquercitrin, rutin, and quercetin in AF [14–18]. Wang et al. [19] used gas chromatography–mass spectrometry (GC–MS) to analyze the volatile oil components of AF extracted by water distillation and liquid–liquid extraction. The existing literature only reported the volatile components of AF from a single producing region and lacked scientific analysis methods and comparison of different producing regions. At present, there were no comprehensive analysis and comparison of volatile components of AF from different producing regions. Headspace (HS) injection was a convenient and fast sample pretreatment method for the GC method. The traditional method to detect the volatile components of the sample was to extract and analyze components of the volatile oil. The HS method has an advantage in the sampling mode compared with the traditional methods, which simplifies the sampling method and saves time [20].

TCM had the characteristics of multicomponents, multitargets, and multipathways acting on the human body. Network pharmacology connects components, targets, and pathways to form a topological network diagram [21]. Researchers have found that the extract of AF had an obvious antidepressant effect by studying the changes in the mental behavior of mice in the model of depression [22]. Xiong et al. [23] constructed a component–target–pathway network of AF, and molecular docking of active components and their principal targets also confirmed the flavonoids quercetin, rutin, and isorhamnetin with a range of pharmacological effects, including sedation, hypnosis, and antidepressant. In modern pharmacological studies, the antidepressant mechanism of flavonoids in AF was mainly to reduce oxidative stress, inhibit inflammation, protect hippocampal neurons, and inhibit monoamine oxidase activity. The volatile constituents of AF significantly contributed to its pharmacological efficacy. Nonetheless, there remained a paucity of research in this domain.

**FIGURE 1:**
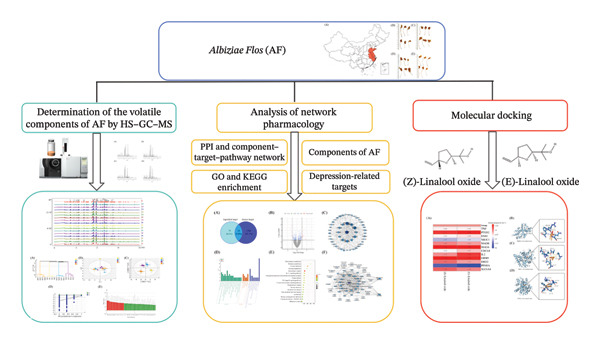
Schematic diagram of exploration of potential antidepressant active ingredients of *Albiziae Flos* via HS–GC–MS, chemometrics, and network pharmacology.

In this study, headspace–gas chromatography–mass spectrometry (HS–GC–MS) was applied to evaluate the volatile components of AF from various production regions, and the similarities and differences among AF were assessed using fingerprint and chemometrics analysis. It served as a reference for volatile components and the quality evaluation of AF. Simultaneously, network pharmacology was employed to develop the component–target–pathway diagram illustrating the possible antidepressant effect of AF, and the active components were molecularly docked with critical targets to validate the antidepressant effectiveness of the volatile components of AF (Figure 1). This study provided a reference for the volatile components of AF to alleviate depression and insomnia symptoms.

## 2. Experiments

### 2.1. Materials

A total of 16 batches (S1–S16) of AF samples were purchased from the native Chinese medicine market and drug retail stores of Zhejiang (S1–S4), Jiangsu (S5–S8), Shandong (S9–S12), and Anhui (S13–S16) Provinces in China (Figure 2) and deposited in a cool and dry place at the Innovative Pharmaceutical Excipients Analysis Center of Chongqing Medical University. All AF samples were authenticated as dry AF by Associate Professor Wang Jian (College of Traditional Chinese Medicine, Chongqing Medical University, Chongqing). The reference substance of the oxidized linalool mixture (batch number: L862898, GC purity ≥ 97%) was purchased from McLean.

FIGURE 2Botanical pictures and sampling origin distribution map of AF from different producing regions. (a) AF sample origin distribution map. (b) Zhejiang. (c) Jiangsu. (d) Shandong. (e) Anhui.

**Figure:**
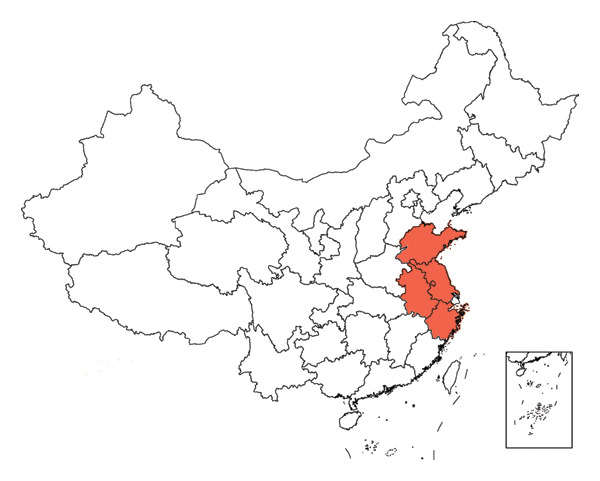
(a)

**Figure:**
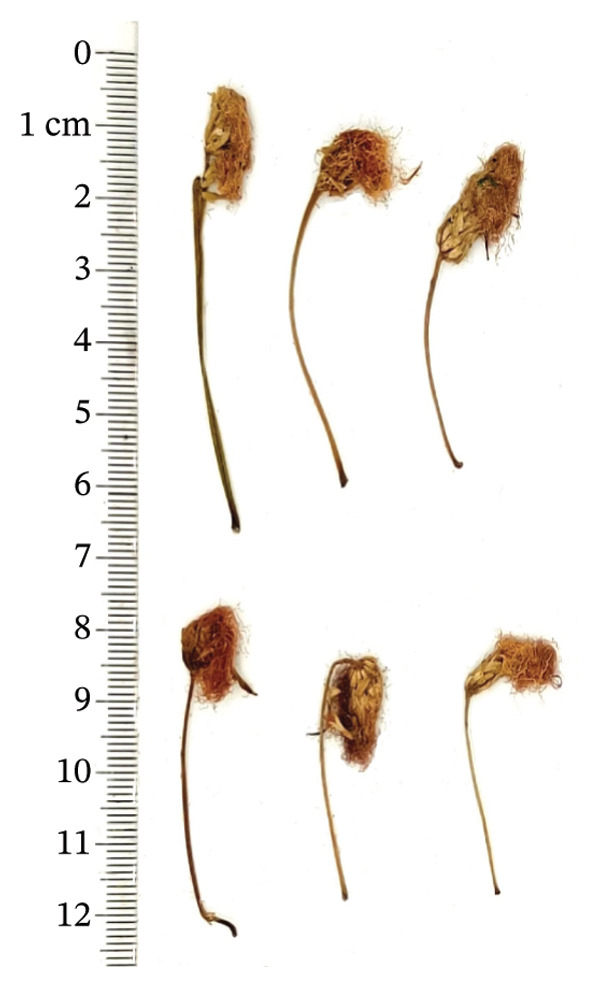
(b)

**Figure:**
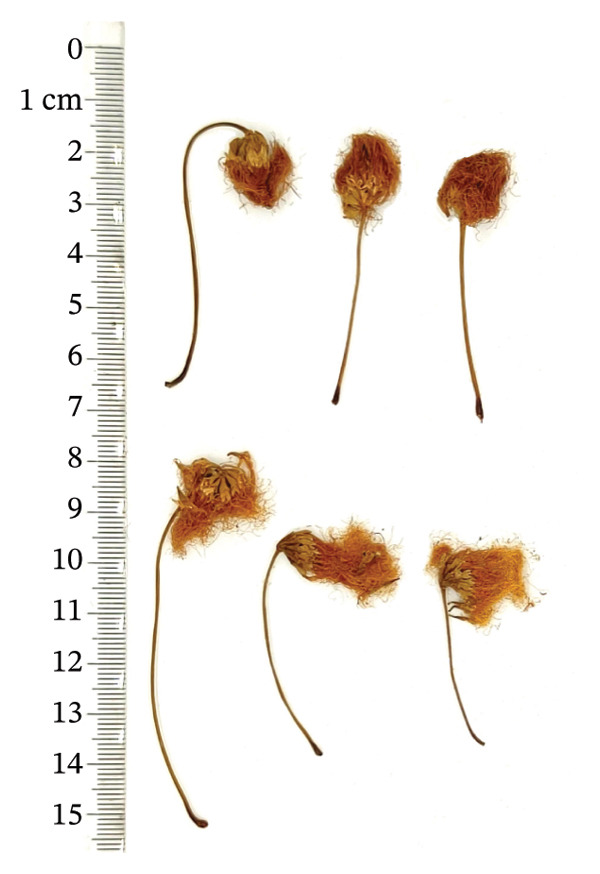
(c)

**Figure:**
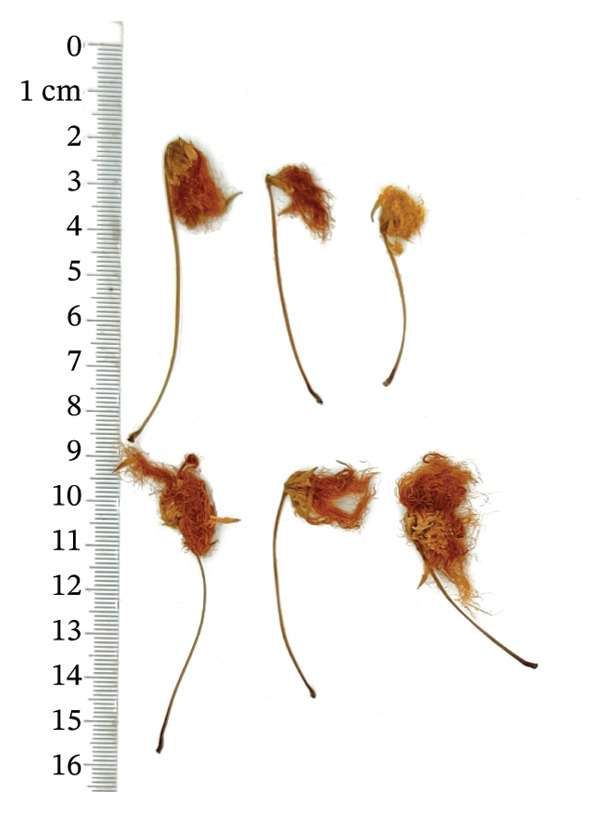
(d)

**Figure:**
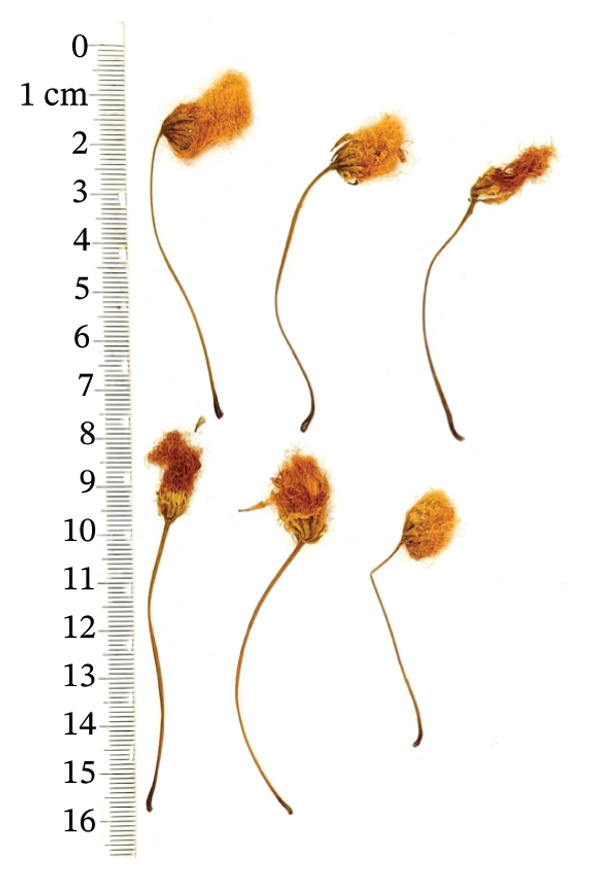
(e)

### 2.2. Methods

#### 2.2.1. Detection and Analysis of Volatile Components in AF

##### 2.2.1.1. HS–GC–MS Condition

Shimadzu TQ8040 GC–MS (Shimadzu, Kyoto, Japan) with an Agilent DB‐17MS column (30 m × 0.25 mm, 0.25 μm) (Agilent, Santa Clara, CA, USA) was used for HS–GC–MS analysis. An LS 220A 1/10,000 electronic balance was obtained from Precisa International Instrument Co., Ltd. (Shanghai, China) and applied for sample weighting. A high‐speed multifunctional grinder (RRH‐A500, Shanghai Yuanwo Industry & Trade Co., Ltd.) was used to crush AF. Other analytical reagents were obtained from Chuandong Chemical Group Co., Ltd. (Chongqing, China).

AF samples were pulverized and passed through an 80‐mesh sieve. AF powder (1.6 g) was accurately measured and placed into a 20‐mL HS vial, with 130°C of incubation temperature and 25 min of incubation time. The quantitative ring temperature was 140°C, the magnetic stirrer speed was 250 rpm, and the injection volume was 1 mL, respectively. The GC conditions were as follows: High‐purity helium (purity > 99.999%) was the carrier gas at a flow rate of 1.0 mL × min^−1^. The injector temperature was 250°C in a split mode (10:1). The column temperature was initially set at 40°C, increased to 100°C at 5°C × min^−1^, and increased to 240°C at 20°C × min^−1^, keeping for 5 min. For MS conditions, the EI source temperature was 250°C, and the electron bombardment energy was 70 eV. The time of solvent delay was 3 min, and the mass scan range was from m/z 45 to 500 in the full scan mode.

##### 2.2.1.2. GC–MS Fingerprint Analysis and Chemometrics Analysis

The volatile components of AF samples were identified by comparing the NIST14s.lib and NIST14.lab mass spectrometry databases. Similarity assessment was conducted utilizing the Similarity Evaluation System for Chromatographic Fingerprint of Traditional Chinese Medicine (2012 edition). The HS–GC–MS data from 16 batches of the AF were analyzed using the Similarity Evaluation System for Chromatographic Fingerprint of Traditional Chinese Medicine (2012 edition) to search for the common peaks and evaluate the similarity. The chromatogram of the S1 sample served as the reference chromatogram (R), with a temporal window width of 0.1. The common mode of chromatogram was produced using automatic matching and Mark peak matching following multipoint correction, whereas the control chromatogram was derived from the median. The acquired data via standardization were imported into SIMCA14.1 software (Umetrics) for chemometric analysis involving principal components analysis (PCA), orthogonal partial least squares–discriminant analysis (OPLS–DA), and hierarchical clustering analysis (HCA).

#### 2.2.2. Analysis of Network Pharmacology

##### 2.2.2.1. Identification of Active Ingredients and Disease Targets

The common volatile components of AF from 16 producing regions and the active components of AF searched by the HERB database and the TCMIP database were input into the PubChem database to obtain the compound structures. The structures were imported into SwissADME database to ascertain the number of hydrogen bond donors (≤ 5), the number of hydrogen bond acceptors (≤ 10), relative molecular mass (≤ 500), lipid–water partition coefficient (≤ 5), the number of rotatable bonds (≤ 10), and additional compound information to determine whether it conformed to the five principles of drug‐like [24, 25]. The TCMSP database and the Swiss Target Prediction database were used to retrieve the known targets of the compounds. The UniProt database converted all the targets into genes and summarized and removed duplicate genes. The GeneCards database was used to search for related targets with “depressive disorder,” “sleep disorder,” and “insomnia” as search terms, and the targets were screened with a relevance score > 10 as the standard. Venny2.1.0 was used to obtain the intersection of component targets and disease targets, and finally, the intersection targets were obtained. A depression dataset GSE12654 was obtained from the GPL8300 platform [26] in the NCBI GEO database, comprising 50 samples of the prefrontal cortex of the brain. We applied the GEO database GEO2R online program (/geo/geo2r/) to analyze and compare samples of normal controls and patients with depression. Volcano maps were generated using the expression of differential genes.

##### 2.2.2.2. Network Analysis of Target Proteins and Protein–Protein Interactions (PPIs)

The obtained target proteins were imported into the STRING.11.0 website, and the species was selected as “Human sapiens.” The intermediate confidence protein interaction parameter score was > 0.40, the unconnected target was hidden, and other parameters were set unchanged to obtain the PPI network. The “Network Analyzer” function in Cytoscape 3.10 (Bethesda Softworks, USA) software was used to analyze the topological properties of the PPI network. Targets with degree values > 10 were selected as potential core targets for subsequent analysis.

##### 2.2.2.3. Analysis of GO and KEGG Enrichment

GO function enrichment analysis and KEGG pathway enrichment analysis were performed on 13 potential target proteins using the DAVID 6.8 database. GO functional enrichment analysis included three parts: biological process (BP), cellular component (CC), and molecular function (MF). KEGG functional analysis was mainly used to obtain signal pathways enriched by potential targets.

##### 2.2.2.4. Construction of Herb–Component–Target–Pathway Network

To better understand the effectiveness of the components, the identified 13 targets, 15 active ingredients, and 18 pathways were imported into Cytoscape 3.10 software to construct the component–target–pathway network diagram.

#### 2.2.3. Molecular Docking of Key Components and Core Targets

Essential and potential components were chosen for molecular docking to confirm the interaction between targets and components [27, 28]. The percentage of (Z)‐linalool oxide and (E)‐linalool oxide in the GC–MS chromatogram of volatile components of the AF was higher, and two configurations of linalool oxide (TCLO) were also used as a key component in network pharmacology. At the same time, AF had the pharmacological effect of depression treatment. Referring to the reports, the TCLO has a therapeutic effect on central diseases [29]. Therefore, the effects of the TCLO on these potential targets were analyzed by molecular docking of linalool oxide with 13 potential protein targets with a network node (Degree value) > 10. Molecular docking validation was conducted with the AutoDock software to determine the minimum binding energy between the principal target protein and the candidate component. Ultimately, PyMOL software and the Protein–Ligand Interaction Profiler website were used to visually determine the binding mode with the lowest binding energy.

#### 2.2.4. Database and Software

NIST14s.lib, NIST14.lab, HERB database, TCMIP database, PubChem database, SwissADME database, TCMSP database, UniProt database, Gene Cards database, DAVID 6.8 database; SIMCA14.1 software (Umetrics), STRING.11.0, Cytoscape 3.10, AutoDock software, PyMOL software, and Protein‐Ligand Interaction Profiler website.

## 3. Results and Discussion

### 3.1. Analysis of Volatile Components in AF

The volatile components in AF were examined to generate the total ion chromatogram (Figure 3). According to the relevant database, 34 volatile components (Table 1) with similarity greater than 80% were identified from different producing regions, including organic acids (4), esters (1), aldehydes (7), nitrogen‐containing heterocycles (10), alcohols (4), ketones (3), olefins (3), and alkanes (2). According to the characteristic peak charge–mass ratio, retention time, and peak area information, the relative percentage composition of each component was determined using the area normalization approach. A total of 34 components were detected in the samples from four production regions, but variations in composition and content were seen among various batches within the same region. A total of 17 common components were discovered across 16 batches, with (Z)‐linalool oxide, (E)‐linalool oxide, and (3R, 6S)‐2,2,6‐trimethyl‐6‐vinyltetrahydro‐2H‐pyran‐3‐ol showing the highest relative percentages. (Z)‐Linalool oxide, (E)‐linalool oxide [30], alpha‐terpineol [31], and eicosane [4] have been documented in the literature. Variations in component composition and content among batches from the same producing region implied that environmental factors [32] (e.g., soil conditions, climate) and postharvest handling may exert non‐negligible impacts on AF volatile profiles. The detection of documented components including α‐terpineol and eicosane aligned with previous reports, verifying the reliability of our HS–GC–MS analytical method. Notably, the presence of linalool oxide isomers, known for their aromatic and potential bioactive properties, also offered preliminary clues for investigating the correlation between AF volatile components and its pharmacological effects, such as antidepressant activity [33].

FIGURE 3Total ion chromatograms of volatile components of AF from different producing regions. (a) Zhejiang. (b) Jiangsu. (c) Shandong. (d) Anhui. 1. Acrylic acid. 2. n‐Hexanal; 3. Pyridine; 4. 1‐Hexanol; 5. 2‐Methylpyrazine; 6. 2‐Heptanone; 7. Heptaldehyde; 8. 2,5‐Dimethylfuran; 9. Hexanoic acid; 10. 3‐(1‐Methylpropyl)‐cyclohexene; 11. (+)‐limonene; 12. Octanal; 13. 2,5‐Dimethyl‐2‐vinyl‐1,4‐hexadiene; 14. Benzaldehyde; 15. 1‐Piperidinecarbonitrile; 16. (Z)‐Linalool oxide; 17. (E)‐Linalool oxide; 18. Nonanal; 19. Hotrienol; 20. 2‐Pyrrolecarbaldehyde; 21. 2‐Phenylethanal; 22. 2‐Acetylpyrrole; 23. Tridecane; 24. n‐Decanal; 25. (3R,6S)‐2,2,6‐Trimethyl‐6‐vinyltetrahydro‐2H‐pyran‐3‐ol; 26. alpha.‐Terpineol; 27. 4H‐Pyran‐4‐one, 3‐hydroxy‐2‐methyl‐; 28. Nonanoic acid; 29. Dipentene dioxide; 30. 2‐Methoxy‐4‐vinyl phenol; 31. Cedrenol; 32. 2,6,10‐trimethyl‐14‐pentadecanone; 33. n‐Hexadecanoic acid; 34. Eicosane.

**Figure:**
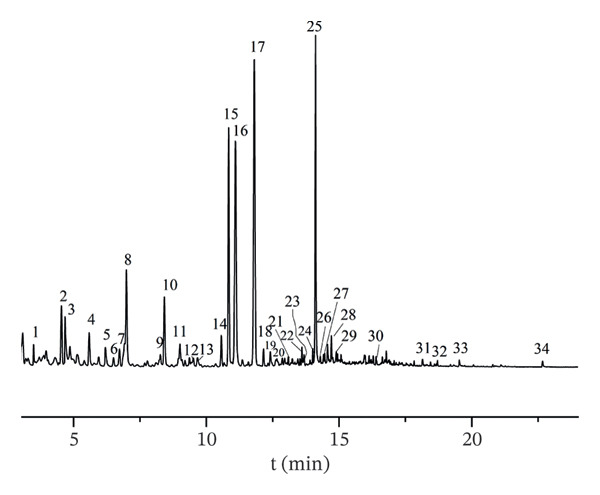
(a)

**Figure:**
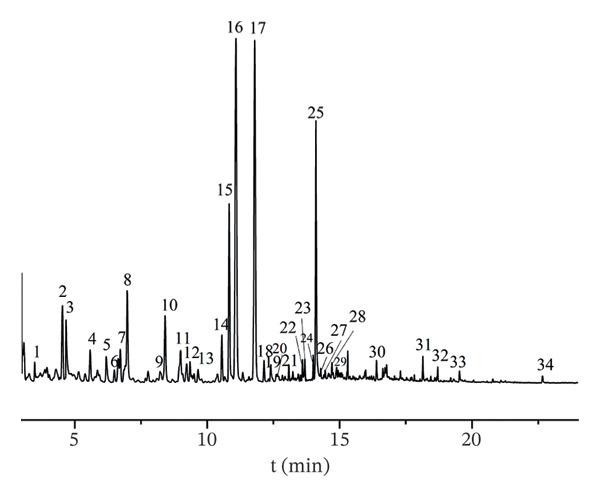
(b)

**Figure:**
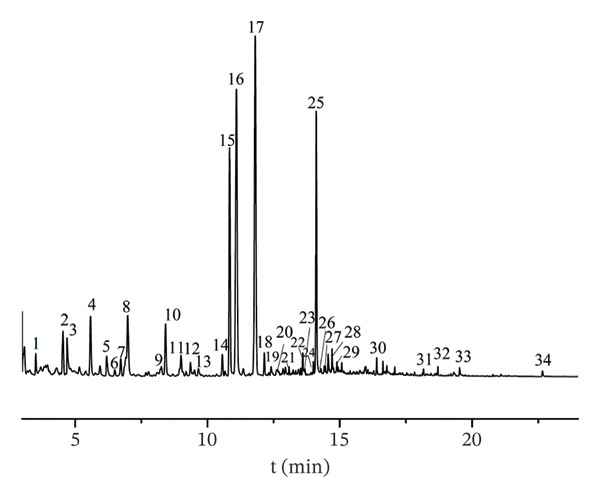
(c)

**Figure:**
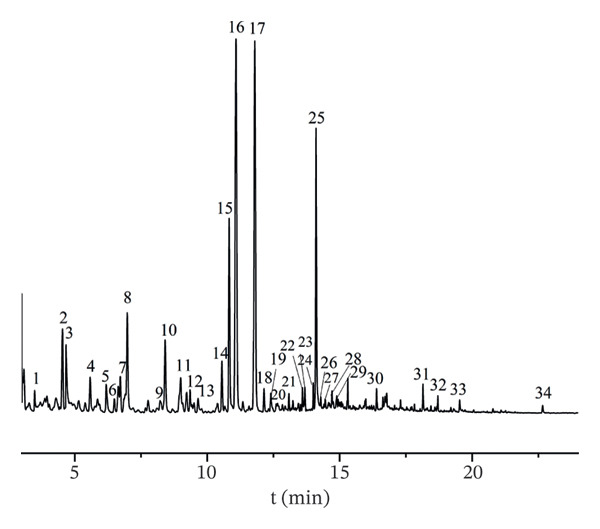
(d)

**TABLE 1:** Qualitative and relative quantitative analysis of volatile components of the AF from different producing regions.

No.	Retention time	Compound	Molecular formula	CAS number	Peak areas (%)			
					Zhejiang	Jiangsu	Shandong	Anhui
1	3.525	Acrylic acid	C_3_H_4_O_2_	79‐10‐7	0.43 ± 0.24	0.30 ± 0.30^∗^	0.58 ± 0.32	0.45 ± 0.26
2	4.540	n‐Hexanal	C_6_H_12_O	66‐25‐1	2.97 ± 0.37	2.32 ± 1.05	2.70 ± 1.23	2.35 ± 1.19
3	4.680	Pyridine	C_5_H_5_N	110‐86‐1	174 ± 1.74^∗^	2.22 ± 1.42	1.74 ± 0.34	1.76 ± 0.74
4	5.585	1‐Hexanol	C_6_H_14_O	111‐27‐3	1.84 ± 0.78	1.40 ± 1.07	3.35 ± 2.96	1.12 ± 0.46
5	6.200	2‐Methylpyrazine	C_5_H_6_N_2_	109‐08‐0	1.02 ± 0.31	0.91 ± 1.53	1.02 ± 0.54	0.76 ± 0.44
6	6.505	2‐Heptanone	C_7_H_14_O	110‐43‐0	0.24 ± 0.24^∗^	0.01 ± 0.37^∗^	0.31 ± 0.31^∗^	0.25 ± 0.25^∗^
7	6.735	Heptaldehyde	C_7_H_14_O	111‐71‐7	1.02 ± 0.22	0.81 ± 0.76	0.86 ± 0.61	0.94 ± 0.63
8	6.985	2,5‐Dimethylfuran	C_6_H_8_O	625‐86‐5	1.90 ± 1.36	1.48 ± 1.36	2.82 ± 1.59	3.29 ± 2.16
9	8.270	Hexanoic acid	C_6_H_12_O_2_	142‐62‐1	0.14 ± 0.19^∗^	0.15 ± 0.45^∗^	0.35 ± 0.35^∗^	0.31 ± 0.31^∗^
10	8.425	3‐(1‐Methylpropyl)‐Cyclohexene	C_10_H_18_	15,232‐91‐4	2.80 ± 1.07	2.18 ± 0.95	3.9 ± 1.54	2.44 ± 0.98
11	8.995	(+)‐limonene	C_10_H_16_	5989‐54‐8	1.47 ± 0.87	0.65 ± 1.69^∗^	0.08 ± 0.24^∗^	1.34 ± 1.34^∗^
12	9.375	Octanal	C_8_H_16_O	124‐13‐0	0.53 ± 0.19	0.28 ± 0.28^∗^	0.55 ± 0.38	0.75 ± 0.36
13	9.665	2,5‐Dimethyl‐2‐vinyl‐1,4‐hexadiene	C_10_H_16_	2153‐66‐4	0.17 ± 0.51^∗^	0.39 ± 0.39^∗^	0.37 ± 0.37^∗^	0.51 ± 0.09
14	10.580	Benzaldehyde	C_7_H_6_O	100‐52‐7	1.32 ± 0.69	0.57 ± 1.08^∗^	1.00 ± 0.71	1.01 ± 0.93
15	10.830	1‐Piperidinecarbonitrile	C_6_H_10_N_2_	1530‐87‐6	8.44 ± 3.29	5.58 ± 4.01	10.67 ± 7.25	6.53 ± 4.86
16	11.105	(Z)‐Linalool oxide	C_10_H_18_O_2_	5989‐33‐3	19.34 ± 7.61	27.96 ± 10.6	22.79 ± 17.2	29.18 ± 7.73
17	11.825	(E)‐Linalool oxide	C_10_H_18_O_2_	34,995‐77‐2	23.75 ± 6.71	28.01 ± 5.36	24.13 ± 8.94	25.34 ± 4.70
18	12.170	Nonanal	C_9_H_18_O	124‐19‐6	0.83 ± 0.15	0.81 ± 0.23	1.04 ± 0.50	0.87 ± 0.17
19	12.415	Hotrienol	C_10_H_16_O	20,053‐88‐7	0.29 ± 0.29^∗^	0.11 ± 0.32^∗^	0.26 ± 0.26^∗^	0.34 ± 0.43^∗^
20	12.615	2‐Pyrrolecarbaldehyde	C_5_H_5_NO	1003‐29‐8	0.18 ± 0.11^∗^	0.33 ± 0.36^∗^	0.14 ± 0.40^∗^	0.12 ± 0.37^∗^
21	13.085	2‐Phenylethanal	C_8_H_8_O_1_	122‐78‐1	0.29 ± 0.03	0.29 ± 0.11	0.26 ± 0.10	0.38 ± 0.12
22	13.600	2‐Acetylpyrrole	C_6_H_7_NO	1072‐83‐9	0.56 ± 0.11	0.37 ± 0.46^∗^	0.71 ± 0.16	0.35 ± 0.35^∗^
23	13.680	Tridecane	C_13_H_28_	629‐50‐5	0.40 ± 0.09	0.18 ± 0.05	0.43 ± 0.44	0.39 ± 0.37
24	14.010	n‐Decanal	C_10_H_20_O	112‐31‐2	0.38 ± 0.18	0.33 ± 0.21	0.32 ± 0.12	0.38 ± 0.21
25	14.110	(3R,6S)‐2,2,6‐Trimethyl‐6‐vinyltetrahydro‐2H‐pyran‐3‐ol	C_10_H_18_O_2_	39,028‐58‐5	14.34 ± 2.39	10.65 ± 4.61	9.77 ± 2.79	9.34 ± 1.92
26	14.305	alpha.‐Terpineol	C_10_H_18_O	98‐55‐5	0.46 ± 0.16	0.14 ± 0.15^∗^	0.19 ± 0.19^∗^	0.35 ± 0.03
27	14.475	4H‐Pyran‐4‐one, 3‐hydroxy‐2‐methyl‐	C_10_H_18_O_2_	118‐71‐8	0.12 ± 0.17^∗^	0.33 ± 0.86^∗^	0.03 ± 0.08^∗^	0.08 ± 0.12^∗^
28	14.735	Nonanoic acid	C_9_H_18_O_2_	112‐05‐0	0.42 ± 0.18	0.36 ± 0.17	0.78 ± 0.60	0.27 ± 0.37
29	14.889	Dipentene dioxide	C_6_H_6_O_4_	96‐08‐2	0.26 ± 0.13	0.35 ± 0.11	0.42 ± 0.17	0.35 ± 0.09
30	16.400	2‐Methoxy‐4‐vinyl phenol	C_9_H_10_O_2_	7786‐61‐0	0.39 ± 0.18	0.39 ± 0.34	0.34 ± 0.23	0.28 ± 0.23
31	18.150	Cedrenol	C_15_H_24_O	28,231‐03‐0	0.53 ± 0.67^∗^	0.24 ± 0.39^∗^	0.04 ± 0.10^∗^	0.35 ± 0.77^∗^
32	18.715	2,6,10‐Trimethyl‐14‐pentadecanone	C_18_H_36_O	502‐69‐2	0.04 ± 0.11^∗^	0.13 ± 0.13^∗^	0.10 ± 0.17^∗^	0.05 ± 0.16^∗^
33	19.530	n‐Hexadecanoic acid	C_16_H_32_O_2_	57‐10‐3	0.11 ± 0.17^∗^	0.23 ± 0.40^∗^	0.09 ± 0.13^∗^	0.13 ± 0.13^∗^
34	22.685	Eicosane	C_20_H_42_	112‐95‐8	0.10 ± 0.10^∗^	0.13 ± 0.19^∗^	0.13 ± 0.17^∗^	0.06 ± 0.17^∗^

^∗^Some samples from the same producing region were not detected.

### 3.2. GC–MS Fingerprint Analysis and Chemometrics Analysis

#### 3.2.1. GC–MS Fingerprint Analysis

**FIGURE 4:**
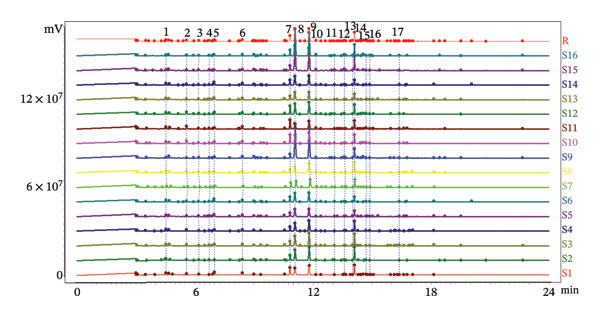
GC–MS fingerprint of volatile components in the AF from different habitats.

The GC fingerprint of AF was established. The similarity assessment results of 16 batches of AF via HS–GC–MS (Figure 4) showed that a total of 17 common peaks were identified, and the similarity of 16 batches of samples was between 0.790 and 0.998, signifying a high degree of similarity in the volatile components. The RSD of retention time was 0.02–1.55%, indicating that the peak time of the common volatile components of different batches of AF was relatively close, and the RSD of peak area was 8.37–71.42%, suggesting that the content of the common volatile components of AF from different producing regions was quite different. The observed variations in volatile component composition and content among intraorigin batches of AF underscore the critical role of extrinsic factors in shaping the herbal material’s chemical profile. Environmental factors and postharvest processing procedures (e.g., drying temperature, storage duration) were plausible drivers of such batch‐to‐batch heterogeneity, a phenomenon that poses challenges for the consistent quality evaluation of AF [34]. This finding highlighted the urgency of establishing standardized cultivation and postharvest processing to minimize chemical variability, which was essential for ensuring the reproducibility of its clinical efficacy [35]. To further explore the difference and consistency of volatile components in different producing regions, chemometrics was further used to analyze the differences among producing regions.

#### 3.2.2. Chemometrics Analysis

##### 3.2.2.1. HCA

HCA is a widely employed approach for hierarchical clustering of samples within a dataset to ascertain the similarities among the samples [36]. The data were imported into SIMCA14, and HCA was performed to select a single linkage to calculate the distance and height to describe. The results (Figure 5(a)) demonstrated that the 16 batches were classified into two categories: SD origin of the AF as one class and ZJ, JS, and AH origins of AF as another class. SD origin was given priority to mountain area, mild climate, and moderate rainfall; ZJ, JS, and SD origins were mild and humid climate; and mountain region is more suitable for the growth of the AJD than the plain. When the ordinate was 3, the four producing regions could be divided into four categories, indicating that the volatile components of the AF have little difference in producing regions.

FIGURE 5Chemometric analysis of AF from different habitats. (a) HCA tree plot; (b) PCA score scatter plot; (c) OPLS–DA score scatter plot; (d) 200 permutation test plot; (e) VIP plot. 1. Acrylic acid. 2. n‐Hexanal; 3. Pyridine; 4. 1‐Hexanol; 5. 2‐Methylpyrazine; 6. 2‐Heptanone; 7. Heptaldehyde; 8. 2,5‐Dimethylfuran; 9. Hexanoic acid; 10. 3‐(1‐Methylpropyl)‐Cyclohexene; 11. (+)‐limonene; 12. Octanal; 13. 2,5‐Dimethyl‐2‐vinyl‐1,4‐hexadiene; 14. Benzaldehyde; 15. 1‐Piperidinecarbonitrile; 16. (Z)‐Linalool oxide; 17. (E)‐Linalool oxide; 18. Nonanal; 19. Hotrienol; 20. 2‐Pyrrolecarbaldehyde; 21. 2‐Phenylethanal; 22. 2‐Acetylpyrrole; 23. Tridecane; 24. n‐Decanal; 25. (3R,6S)‐2,2,6‐Trimethyl‐6‐vinyltetrahydro‐2H‐pyran‐3‐ol; 26. alpha.‐Terpineol; 27. 4H‐Pyran‐4‐one, 3‐hydroxy‐2‐methyl‐; 28. Nonanoic acid; 29. Dipentene dioxide; 30. 2‐Methoxy‐4‐vinyl phenol; 31. Cedrenol; 32. 2,6,10‐trimethyl‐14‐pentadecanone; 33. n‐Hexadecanoic acid; 34. Eicosane.

**Figure:**
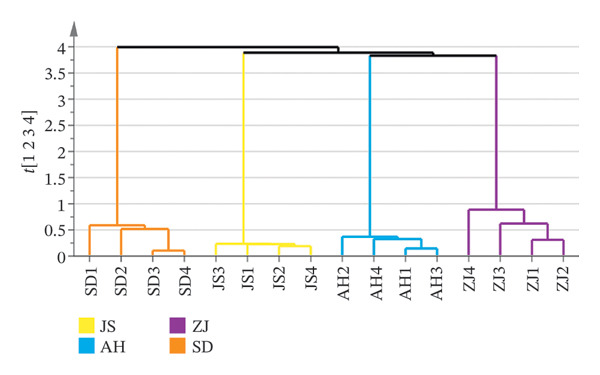
(a)

**Figure:**
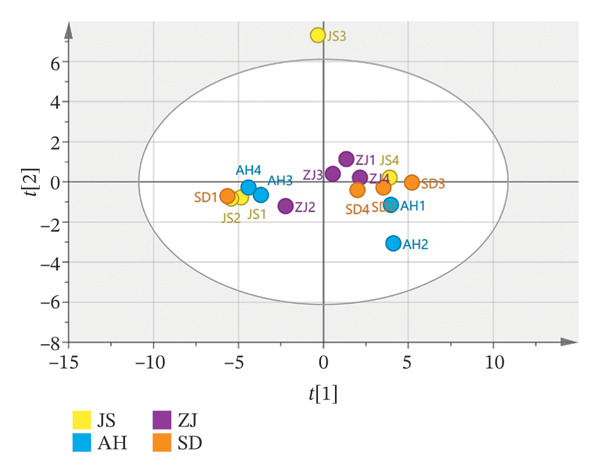
(b)

**Figure:**
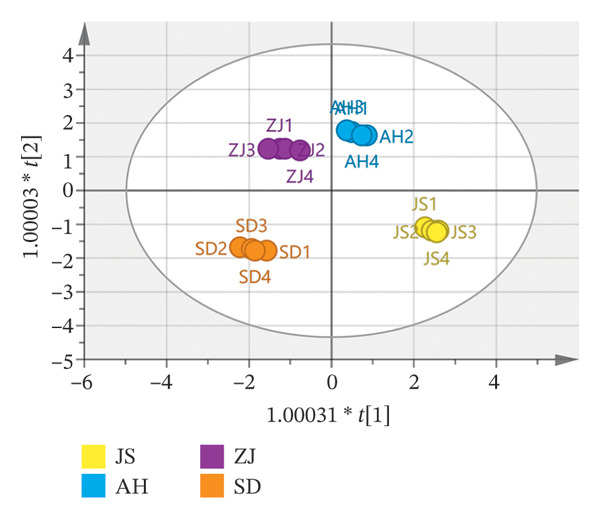
(c)

**Figure:**
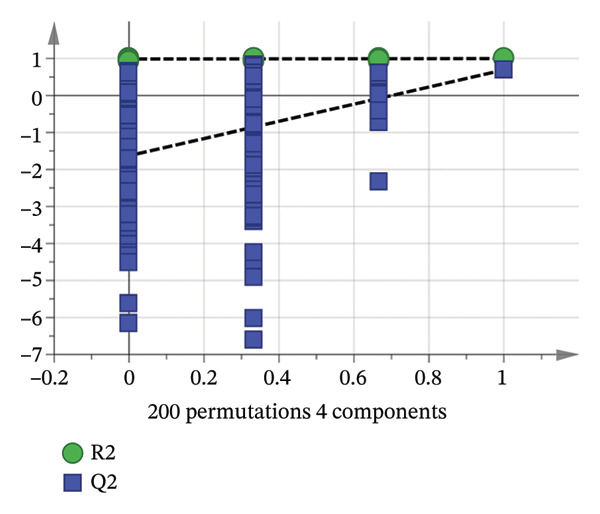
(d)

**Figure:**
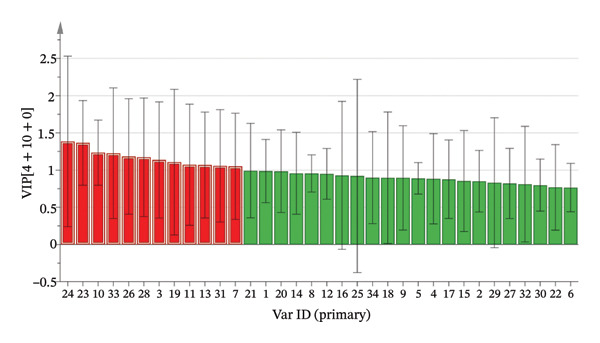
(e)

##### 3.2.2.2. PCA

PCA can reduce the data dimension of a large number of interrelated original data sets and visually display them to reflect the correlation between the original variables. It was an unsupervised method for exploratory data analysis tools, which can be used for visualization, data compression, checking groups and trends in data, detecting outliers, etc. [37, 38]. According to the results of the fingerprint, the differences of volatile components in different batches and producing regions of AF and the relative content of volatile components were also quite different. Therefore, the responsivity of 34 peak components in 16 batches was introduced into SIMCA14.1 for chemometric analysis. Firstly, unsupervised PCA was performed. The PCA score scatter plot (Figure 5(b)) indicated that the model parameters *R* ^2^ and *Q* ^2^ were 0.676 and 0.234, respectively, signifying that 67.6% and 23.4% (*R* ^2^ ≤ 0.9, *Q* ^2^ ≤ 0.5) of the total variables could be elucidated and forecasted, and demonstrating the model’s inadequate predictive capability [39].

##### 3.2.2.3. OPLS–DA

OPLS–DA is a supervised discriminant analysis statistical method that combines orthogonal signal (OSC) and PLS–DA methods. When analyzing the data, the grouping of the samples is known, which can better distinguish the characteristic variables of each group and determine the relationship between the samples [40]. A supervised OPLS–DA was conducted to elucidate the distinctions among AF groups from various production regions and to categorize and forecast these regions. The OPLS–DA score scatter plot (Figure 5(c)) exhibited that the model parameters *R* ^2^ *X*  = 0.993, *R* ^2^ *Y*  = 0.999, *Q* ^2^ = 0.781 (*R* ^2^ > 0.9, *Q* ^2^ > 0.5), reflecting that the model accounted for 99.3% and 99.9% of the *X* and Y matrices, respectively. The model had a prediction accuracy of 78.1%, signifying that the stability and predictive capability of the OPLS–DA model were commendable. The figure clearly delineated the various geographical sources of AF. To verify whether the model had overfitting, 200 permutation tests were performed (Figure 5(d)). The results showed that the intercepts of *R* ^2^ *X* and *Q* ^2^ *Y* are 0.998 and −1.63, respectively, and *R* ^2^ *X* and *Q* ^2^ *Y* are > 0.3 and < 0.05, respectively, indicating that the model has good reliability and persuasiveness [41]. Variable importance projection (VIP) was used to identify the differential markers between different producing regions. VIP diagram (Figure 5(e)) showed that 12 volatile components had a great contribution to the classification of producing regions (VIP value > 1) [42]. The contribution degree from large to small: n‐decanal, tridecane, 3‐(1‐methylpropyl)‐cyclohexene, n‐hexadecanoic acid, alpha.‐terpineol, nonanoic acid, pyridine, hotrienol, (+)‐limonene, 2,5‐dimethyl‐2‐vinyl‐1,4‐hexadiene, cedrenol, and heptaldehyde. It revealed that the above index components can be used as measurement indices when identifying the origin of AF from different producing regions. 1‐Hexanol, nonanoic acid, and (+)‐limonene oxide were also selected as active ingredients in network pharmacology for antidepressant pharmacological analysis. According to the concept of Q‐marker [43] proposed by Academician Liu Changxiao, 1‐hexanol, nonanoic acid, and (+)‐limonene oxide can be used as potential Q‐marker for the quality evaluation of AF.

### 3.3. Network Pharmacology Analysis

#### 3.3.1. Identification of Active Components of AF and Disease Targets

A total of 15 active components were identified based on GC–MS, the HERB database, the TCMIP database, the PubChem database, and the SwissADME database: hexanal, 1‐hexanol, heptanal, 3‐(1‐methyl propyl)‐cyclohexene, 1‐pyridine methyl ester, (S)‐linalool oxide, (E)‐linalool oxide, pelargonaldehyde, phenylacetaldehyde, nonanoic acid, (+)‐limonene oxide, quercitrin, linalool, isoamyl alcohol, and cyanidin‐3‐O‐glucoside. The obtained active ingredients were entered into the TCMSP database and the Swiss Target Prediction database to limit the species to “Homo sapiens” to obtain the component targets. All the targets were converted into genes through the UniProt database, and the duplicate genes were summarized and removed to obtain 131 targets. Using “depressive disorder,” “sleep disorder,” and “insomnia” as keywords, a total of 1821 related target proteins were collected in the GeneCards database. After Venny 2.1.0 software mapping, a total of 53 direct targets for the treatment of disease were obtained (Figure 6(a)). Fifty samples from the brain prefrontal cortex in the GSE12654 dataset were compared and analyzed using the GEO2R online tool from the GEO database (Figure 6(b)). The volcano map could visually see the differences in gene expression in patients with depression. Genes with *p* < 0.05 and | log2 FC | ≥ 1 in the GSE12654 dataset were screened by volcano plot analysis. It was found that OR1E1, WIPF1, ARMC6, SLC28A2, SOX30, FAM13C, and CASP5 genes were significantly downregulated. GPR143, ENPEP, PHACTR4, DKFZP547J0410, ITGB8, CROT, RAB9BP1, and PSPHP1 genes were significantly upregulated. The identification of 15 bioactive components from AF via an integrated strategy combining GC–MS detection and multidatabase mining underscores the complexity of AF’s pharmacodynamic material basis, with linalool oxide isomers and cyanidin‐3‐O‐glucoside emerging as potential core active constituents. The 53 overlapping targets obtained by intersecting component‐related and disease‐associated targets (depressive disorder, sleep disorder, insomnia) served as critical molecular nodes linking AF’s volatile and nonvolatile components to its therapeutic effects, laying a foundation for deciphering the herb’s multitargeted regulatory mechanisms [44].

FIGURE 6Network pharmacology study on antidepressant and insomnia of volatile components in AF. (a) Venn diagram of component target–disease target. (b) Volcano map of DEGs in the GSE12654 dataset. (c) Protein–protein interaction network analysis. (d) GO function enrichment analysis. (e) KEGG enrichment analysis. (f) Herb–component–target–pathway network diagram.

**Figure:**
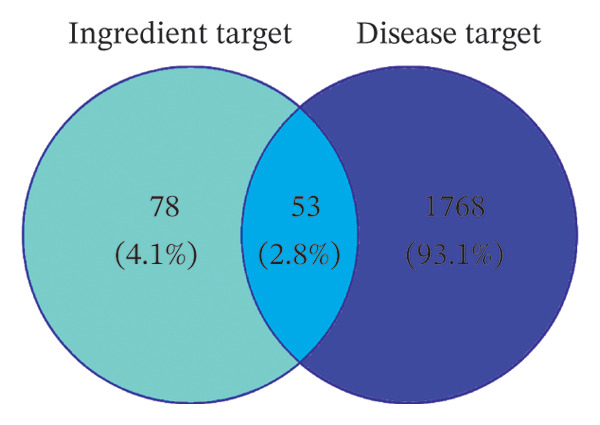
(a)

**Figure:**
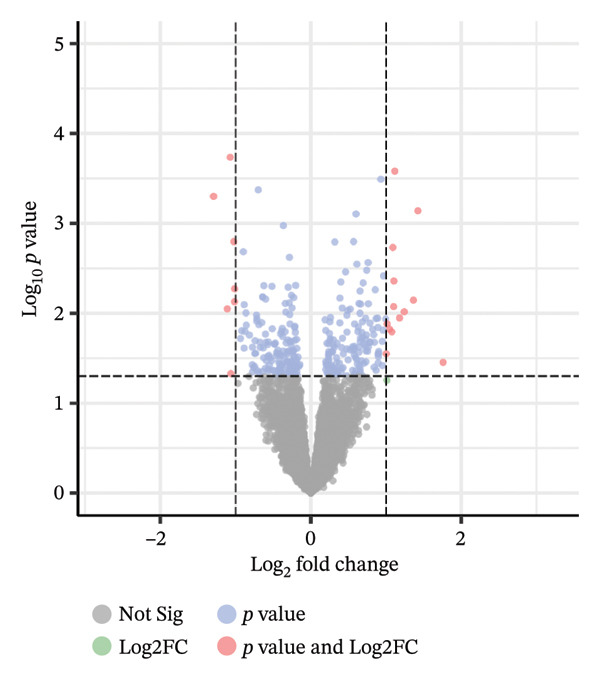
(b)

**Figure:**
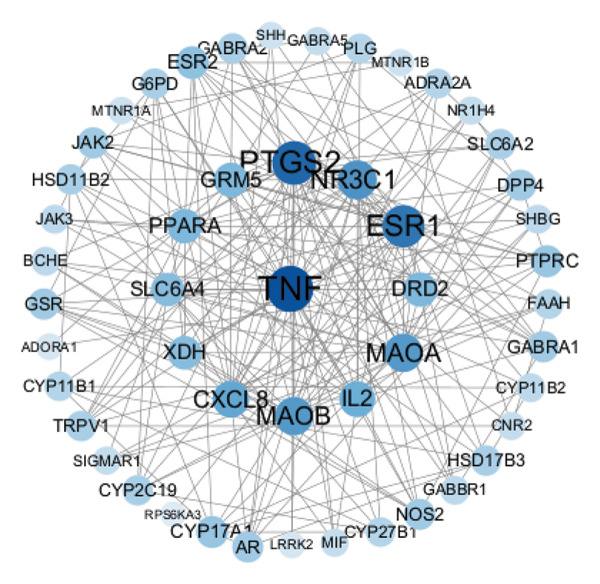
(c)

**Figure:**
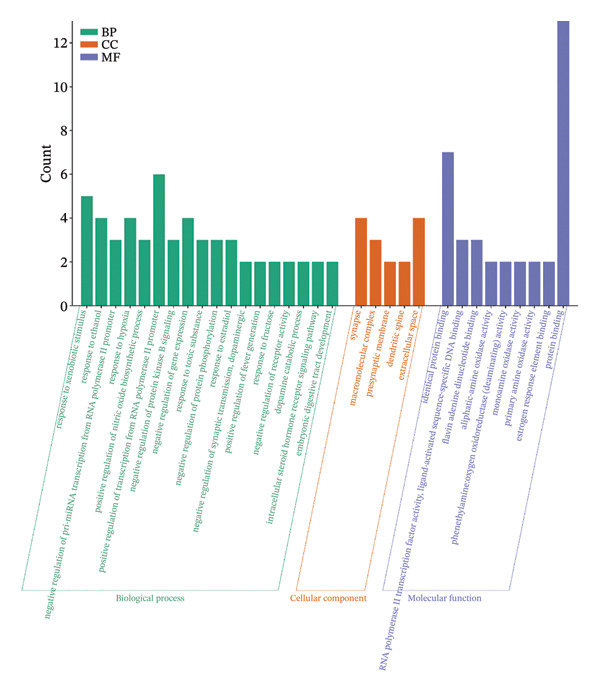
(d)

**Figure:**
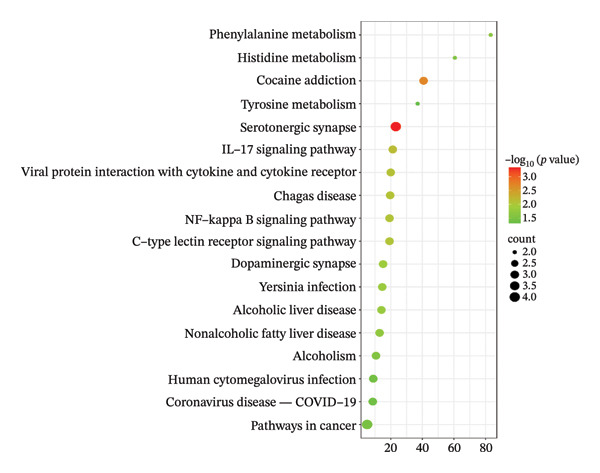
(e)

**Figure:**
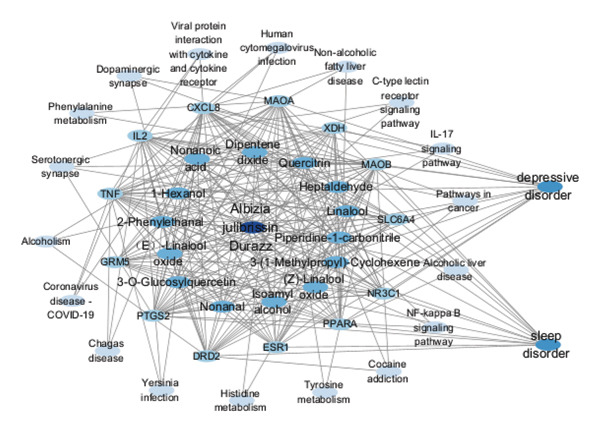
(f)

#### 3.3.2. PPI Analysis

The PPI diagram was generated using STRING.11.0 version and cytospace3.10 version (Figure 6(c)). The genes with degree value exceeding 10 were identified as promising targets (Table 2), likely serving as the key targets for antidepressants and insomnia related to the AF for further investigation.

**TABLE 2:** Degree values of potential core targets.

No.	UniProt ID	Name of the target protein	Gene symbol	Degree value
1	P01375	Tumor necrosis factor	TNF	27
2	P35354	Prostaglandin G/H synthase 2	PTGS2	24
3	P03372	Estrogen receptor alpha	ESR1	22
4	P04150	Glucocorticoid receptor	NR3C1	18
5	P27338	Monoamine oxidase B	MAOB	17
6	P21397	Monoamine oxidase A	MAOA	17
7	P10145	C‐X‐C motif chemokine ligand 8	CXCL8	14
8	P60568	Interleukin 2	IL2	14
9	P41594	Glutamate metabotropic receptor 5	GRM5	12
10	P14416	Dopamine D2 receptor (by homology)	DRD2	12
11	Q07869	Peroxisome proliferator–activated receptor alpha	PPARA	12
12	P31645	Solute carrier family 6 member 4	SLC6A4	11
13	P47989	Xanthine dehydrogenase/oxidase	XDH	11

#### 3.3.3. GO Functional Enrichment Analysis and KEGG Pathway Analysis

A total of 77 findings were derived from GO functional analysis, comprising 56 linked to BPs, 5 pertaining to CCs, and 16 associated with MFs. Based on a *p* value of less than 0.01, 18 results were derived from BP and 9 results were derived from MF. The visual mapping analysis was conducted (Figure 6(d)). Studies have shown that the potential active ingredients of BP were mainly related to response to xenobiotic stimulus, response to ethanol, negative regulation of pri‐miRNA transcription from RNA polymerase II promoter, etc. CC was mainly about synapses, macromolecular complexes, presynaptic membranes, dendritic spines, and extracellular spaces. MF was mainly regarding identical protein binding, RNA polymerase II transcription factor activity, ligand‐activated sequence‐specific DNA binding, etc. KEGG enriched a total of 35 pathways. On the basis of a *p* value of less than 0.05, 18 paths were found, and selected results were displayed (Figure 6(e)). It was mainly about serotonergic synapse, cocaine addiction, IL‐17 signaling pathway, viral protein interaction with cytokine and cytokine receptor, Chagas disease, NF‐kappa B signaling pathway, and C‐type lectin receptor signaling pathway. GO functional enrichment and KEGG pathway analyses provided mechanistic insights into how AF active components modulate depressive disorder and sleep‐related pathologies. BP enrichment results highlight a focus on neurotransmitter regulation and stress‐responsive signaling, which align with the core pathological mechanisms of depression involving neurotransmitter imbalance and neuroplasticity impairment [45]. CC annotations of synapse and presynaptic membrane further emphasize AF’s potential to target neural communication interfaces, while MF terms such as protein binding and transcription factor activity indicate its capacity to regulate gene expression and protein interactions [46]. KEGG pathway enrichment identified key pathways, including serotonergic synapse, NF‐*κ*B signaling, and IL‐17 signaling, which were well‐documented to participate in depressive‐like behaviors: Serotonergic synapse dysfunction was a classic therapeutic target for antidepressants, and NF‐*κ*B/IL‐17 pathways link neuroinflammation to depression pathogenesis. Collectively, these findings illustrated that AF may exert therapeutic effects through a multitarget, multipathway regulatory network, bridging its chemical constituents to biological functions. Future studies should validate these pathways via in vitro and in vivo experiments to confirm AF’s regulatory effects on key targets [23].

#### 3.3.4. Construction of the Herb–Component–Target–Pathway Network

The study performed a comprehensive investigation of medicinal materials, components, diseases, major targets, and signaling pathways to elucidate the efficacy of the components (Figure 6(f)). Depression and insomnia were mainly related to molecules such as dopamine and 5‐hydroxytryptamine secreted by the body [47], so blocking the hydrolysis of these molecules can effectively alleviate depressive symptoms such as depression. The serotonergic synapse signaling pathway can regulate MAOB, MAOA, PTGS2, and SLC6A4 targets; reduce the hydrolysis of molecules such as dopamine and 5‐hydroxytryptamine; increase transport; and enhance the effect. IL‐17 signaling pathway regulates the regulation of CXCL8, PTGS2, and TNF, which can suppress inflammatory factor expression, enhance the transit of neurotransmitters like dopamine and serotonin, and mitigate depressive symptoms [48]. This study showed that potential active ingredients may regulate serotonergic synapse, cocaine addiction, IL‐17 signaling pathway, and other signaling pathways by acting on key targets, such as TNF, PTGS2, ESR1, NR3C1, MAOB, MAOA, CXCL8, IL‐2, and GRM5, and participate in BPs, such as response to a xenobiotic stimulus to alleviate the symptoms of depression and insomnia. Notably, the regulation of the serotonergic synapse pathway by AF components directly addressed the core pathogenesis of depression. By inhibiting the hydrolysis of dopamine and 5‐hydroxytryptamine via MAOB and MAOA, the components elevated synaptic neurotransmitter levels and enhanced neurotransmission [49]. Meanwhile, the suppression of proinflammatory factors through the IL‐17 pathway further mitigated neuroinflammation‐induced synaptic dysfunction, which complements the neurotransmitter‐regulating effect. Collectively, this network framework revealed that AF exerts therapeutic effects through the crosstalk between neurotransmitter homeostasis and neuroinflammation inhibition [50]. Future research should prioritize verifying the binding affinity between key AF components and core targets via molecular docking and in vitro assays to validate the proposed regulatory mechanisms.

### 3.4. Molecular Docking

Through molecular docking of the TCLO with 13 key targets, 12 key targets were successfully docked with linalool oxide, and the binding energy was less than 0. XDH failed to dock with the TCLO (Figure 7(a)). In molecular docking, the binding energy below 0 kcal·mol^−1^ signified that the ligand and the receptor can spontaneously bind, whereas a binding energy less than 5 kcal·mol^−1^ suggested a superior binding affinity between the ligand and receptor. The results of this study revealed that the average binding energy was −4.25 kcal·mol^−1^, with all binding energies beneath −3.34 kcal·mol^−1^, signaling that linalool oxide had a substantial binding affinity for essential target protein molecules. Molecular docking comparison data with fluoxetine, a classic SSRI, are widely used in clinical antidepressant therapy. Specifically, the molecular docking result showed that the binding energy of fluoxetine to the dopamine receptor is −7.9 kcal/mol. Although the average binding energy of TCLO (−4.25 kcal/mol) was slightly higher than that of fluoxetine, it still met the criterion of spontaneous ligand–receptor binding (binding energy < 0 kcal/mol), demonstrating its potential antidepressant activity [51]. Through the visualizations of molecular docking results (Figures 7(b), 7(c), 7(d)), they were shown that TCLO was mainly combined with key target protein molecules through hydrophobic interaction, and stronger hydrogen bonds would appear in some protein molecules so that TCLO were combined with key target protein molecules stronger. The binding affinity of the TCLO to the receptor varied. The binding energy of (Z)‐linalool oxide was lower than that of (E)‐linalool oxide, and the binding to the receptor was stronger. Computer simulations of TCLO and its primary target proteins could confirm the pharmacological potential of TCLO in treating depression and serve as a reference for the development of antidepressant medications.

FIGURE 7The results of molecular docking of the TCLO with 12 key targets. (a) Molecular docking heat map analysis. (b) NR3C1–(Z)‐linalool oxide (−5.07 kcal·mol^−1^). (c) PPARA–(Z)‐linalool oxide (−5.66 kcal·mol^−1^). (d) PPARA–(E)‐linalool oxide (−6.03 kcal·mol^−1^).

**Figure:**
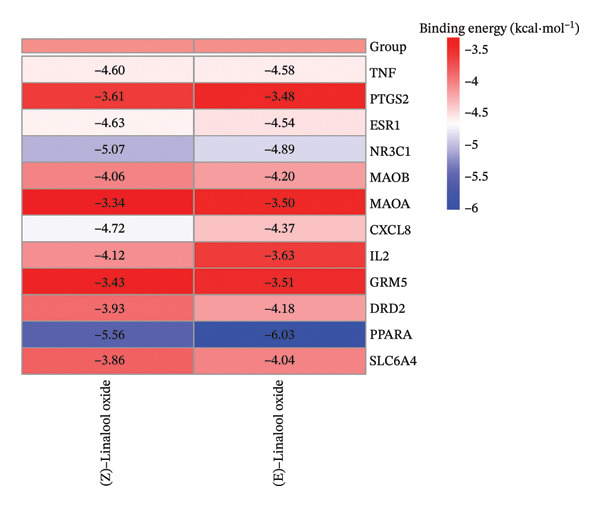
(a)

**Figure:**
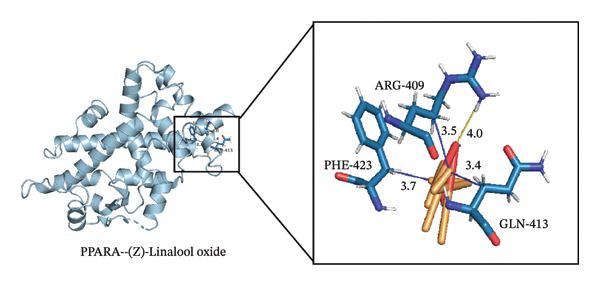
(b)

**Figure:**
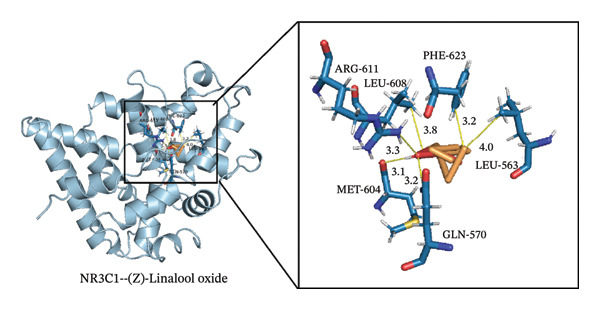
(c)

**Figure:**
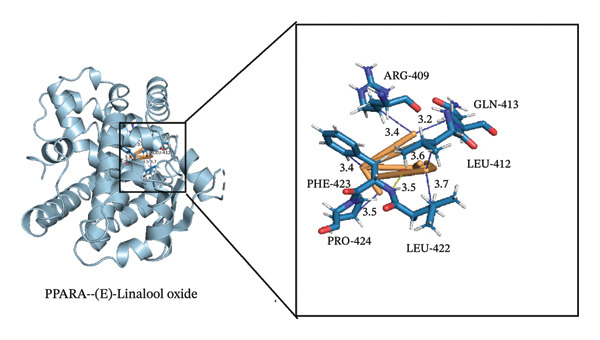
(d)

## 4. Discussion

This study focused on volatile component identification and mechanistic prediction but did not investigate the in vitro/in vivo antidepressant efficacy of AF or its active volatile fractions, limiting the translational value of the findings. To address these gaps, there have outlined clear and actionable future research plans. Firstly, conduct in vitro cell experiments using corticosterone‐induced PC12 cells or primary hippocampal neurons to validate the neuroprotective effects of key volatile components and their regulatory roles in core targets and pathways [52]. Secondly, perform in vivo studies using chronic unpredictable mild stress (CUMS)–induced depressive mice to evaluate behavioral improvements via forced swim test, sucrose preference test, and open field test, while verifying target expression and pathway activation through Western blot, qPCR, and immunohistochemistry [53]. Thirdly, optimize the extraction and purification of active volatile components to explore their potential as lead compounds, and further investigate the synergistic effects of multicomponent combinations [54].

## 5. Conclusions

This study exploited HS–GC–MS technology in combination with chemometrics and network pharmacology. The volatile components of 16 batches of AF from 4 producing regions were identified by HS–GC–MS analysis, and a total of 34 components were identified. Among them, the contents of linalool oxide and (3R, 6S)‐2,2,6‐trimethyl‐6‐vinyltetrahydro‐2H‐pyran‐3‐ol were the highest. The fingerprint revealed 17 shared peaks, with the similarity among 16 batches of the AF ranging from 0.790 to 0.998. Chemometric analysis also distinguished the flowers of AF from different producing regions. In the VIP map, it was also pointed out that 12 volatile components could be used as differential components for the identification of producing regions and could be used as potential Q‐markers. The identified common components of AF and the active components retrieved in the database were used to analyze the antidepressant mechanism of AF by network pharmacology. The results showed that 15 active components such as (S)‐linalool oxide and (E)‐linalool oxide acted on serotonergic synapses and other pathways by regulating key targets such as TNF and PTGS2 to exert the antidepressant effect of AF. At the same time, oxidized linalool was also screened as the active component in network pharmacology. The molecular docking experiment of the TCLO with 13 key targets was carried out to verify the antidepressant network pharmacology of AF. The findings indicated that TCLO effectively interacted with 12 principal targets via hydrogen bonding and hydrophobic interactions, with an average binding energy below −4.25 kcal·mol^−1^. Nonetheless, the potential of TCLO‐enhanced therapeutic outcomes in depression requires additional validation in animal studies.

In summary, this study employed HS–GC–MS in conjunction with chemometrics and network pharmacology to initially identify the active components and principal targets of the volatile constituents of AF in the treatment of depression, thereby offering insights and theoretical guidance for the exploration of TCM in addressing mental disorders like depression.

**Nomenclature**
AF: *Albiziae Flos*
AJD: *Albizia julibrissin Durazz*
TCM: Traditional Chinese medicine
HS–GC–MS: Headspace–gas chromatography–mass spectrometry
TCLO: Two configurations of linalool oxide
BP: Biological process
CC: Cellular component
MF: Molecular function
PPI: Protein–protein interaction
PCA: Principal components analysis
OPLS–DA: Orthogonal partial least squares–discriminant analysis
VIP: Variable importance projection
HCA: Hierarchical clustering analysis

## Author Contributions

Dan Yang: methodology, writing–original draft, and formal analysis. Dan He: writing–review and conceptualization. Yiwu Wang: methodology, writing–original draft, validation, and formal analysis. Yuan Shen: investigation, formal analysis, and validation. Jialing Yu: software and formal analysis. Ruijia Yang: software and formal analysis. Lin Yang: writing–review, conceptualization, and supervision.

## Funding

No funding was received for this manuscript.

## Conflicts of Interest

The authors declare no conflicts of interest.

## Data Availability

The data that support the findings of this study are available from the corresponding author upon reasonable request.
